# Molecular Mechanisms behind the Physiological Resistance to Intense Transient Warming in an Iconic Marine Plant

**DOI:** 10.3389/fpls.2017.01142

**Published:** 2017-06-29

**Authors:** Lazaro Marín-Guirao, Laura Entrambasaguas, Emanuela Dattolo, Juan M. Ruiz, Gabriele Procaccini

**Affiliations:** ^1^Integrative Marine Ecology, Stazione Zoologica Anton DohrnNaples, Italy; ^2^Seagrass Ecology Group, Oceanographic Center of Murcia, Spanish Institute of OceanographyMurcia, Spain

**Keywords:** heat stress, RNA-seq, *Posidonia oceanica*, marine plants, comparative transcriptomics, thermal tolerance, transient warming, mesocosms

## Abstract

The endemic Mediterranean seagrass *Posidonia oceanica* is highly threatened by the increased frequency and intensity of heatwaves. Meadows of the species offer a unique opportunity to unravel mechanisms marine plants activate to cope transient warming, since their wide depth distribution impose divergent heat-tolerance. Understanding these mechanisms is imperative for their conservation. Shallow and deep genotypes within the same population were exposed to a simulated heatwave in mesocosms, to analyze their transcriptomic and photo-physiological responses during and after the exposure. Shallow plants, living in a more unstable thermal environment, optimized phenotype variation in response to warming. These plants showed a pre-adaptation of genes in anticipation of stress. Shallow plants also showed a stronger activation of heat-responsive genes and the exclusive activation of genes involved in epigenetic mechanisms and in molecular mechanisms that are behind their higher photosynthetic stability and respiratory acclimation. Deep plants experienced higher heat-induced damage and activated metabolic processes for obtaining extra energy from sugars and amino acids, likely to support the higher protein turnover induced by heat. In this study we identify transcriptomic mechanisms that may facilitate persistence of seagrasses to anomalous warming events and we discovered that *P. oceanica* plants from above and below the mean depth of the summer thermocline have differential resilience to heat.

## Introduction

Seagrasses are a polyphyletic group of clonal plants of the order Alismatales that have colonized the sea at least on three different occasions along their evolutionary history (Les et al., 1997). In their adaptation to live completely submerged on the marine realm this group of plants converged on a series of structural and physiological modifications, that involved adjustments of their genetic repertoire in respect to terrestrial plants (Wissler et al., 2011; Olsen et al., 2016). Seagrasses lost several characters of the terrestrial counterparts (e.g., absence of stomata) but also gained others typical of macroalgae (e.g., cell wall composition), featuring now clear distinctive attributes and characteristics (Olsen et al., 2016).

Seagrasses are foundation species that strongly influence the structure and function of littoral ecosystems in most coastal areas worldwide. They form extensive meadows that support diverse food webs and provide numerous ecological functions and socio-economical services rendering them one of the most valuable ecosystems on earth (Costanza et al., 2014). This group of marine plants has been experiencing global decline during the last decades (Waycott et al., 2009), and its loss is expected to be aggravated as a consequence of the on-going climate changes (Koch et al., 2013). Mean atmospheric temperatures are gradually increasing and the frequency of extreme short-term heat events is growing with consequences on the structural complexity of seagrass meadows and on the associated ecosystem services (Hoegh-Guldberg and Bruno, 2010). Indeed, in recent years temperate seagrass populations have suffered abrupt deterioration triggered by extreme summer temperatures (Díaz-Almela et al., 2009; Marbá and Duarte, 2010; Moore et al., 2014; Thomson et al., 2015).

In contrast to terrestrial plants, quite little is known about the effects of heating on seagrasses and almost nothing is known about the tolerance mechanisms these can activate to overcome transient exposures to temperature extremes. Understanding heat tolerance mechanisms in seagrasses will be critical to increase our ability to protect and manage these valuable coastal ecosystems and to estimate their plasticity and potential ability to adapt and persist in an ocean that is experiencing unprecedented changes.

Based on the available information, it is generally accepted that in seagrasses mild increases in water temperature lead to an increase in both the photosynthetic and the respiratory rates (Lee et al., 2007; Koch et al., 2013). Although several tropical species have shown an ability to down-regulate respiration (Collier et al., 2011), the rise of respiration is generally proportionally higher than that of photosynthesis (Lee et al., 2007). This can continue over the physiological optimum of the species, where photosynthesis collapses, causing a major metabolic imbalance and dwindling energy reserves, with potential consequences on the ability of plants to survive and recover after transitory thermal stress. At more extreme temperatures, seagrasses can suffer chronic photosynthetic inhibition by direct damage of photosystems II and/or activation of non-photosynthetic quenching processes (Ralph, 1998, 1999; Campbell et al., 2006; Winters et al., 2011; Pedersen et al., 2016). Under short exposures to these temperature extremes seagrass growth is blocked and mortality induced (Campbell et al., 2006; Collier and Waycott, 2014).

Since global warming alters plant performance by directly affecting processes at molecular, biochemical and physiological level, studying these processes under controlled experimental conditions allows to understand the mechanism that would let seagrasses to endure transitory high ambient temperatures (Leakey et al., 2009). Indeed, the ability to measure genome-wide patterns of transcript abundance have provided, in recent years, a new opportunity to improve our understanding of the intrinsic strategies through which seagrasses can respond to global change (Procaccini et al., 2012; Davey et al., 2016). Plastic and adaptive changes of *Zostera marina* and *Z. noltei* to warming have been explored using individuals from contrasting latitudinal origins and exposing them to heat stress under controlled conditions (Franssen et al., 2011, 2014; Gu et al., 2012; Jueterbock et al., 2016). The two species featured responses comparable to terrestrial plants, including the induction of heat shock proteins (HSPs), antioxidant enzymes and proteins involved in cell wall fortification (Gu et al., 2012; Franssen et al., 2014). However, in both species no clear differences in the expression of genes involved in key processes such as photosynthesis, respiration, lipids synthesis and translation/transcription were detected during the heat exposure, despite the differences in growth and photosynthetic thermal tolerances (Franssen et al., 2014). Gene-expression patterns diverged between the populations of different origin during the subsequent recovery, matching their expected differences in heat tolerance (Franssen et al., 2011). This allowed authors to introduce the concept of transcriptomic resilience as a predictor of thermal adaptation in seagrasses, but limited their ability to identify the molecular mechanisms underlying the physiological tolerance of marine plants to transient heat stress.

In the present work, we aim to explore the molecular response mechanisms of an iconic seagrass species during short periods of anomalous warming. *Posidonia oceanica* (L.) Delile is endemic for the Mediterranean Sea and ranks amongst the slowest-growing and longest-lived plants on earth (Arnaud-Haond et al., 2012). The *Posidonia* genus arises from an independent lineage to that of *Zostera* (Les et al., 1997), with which it shows sharp differences in the biological and ecological attributes. *P. oceanica* forms dense and perennial monospecific meadows that extend along a wide bathymetric gradient (from 0 to 40 m depth) all along the Mediterranean coasts, being considered the climax stage of the Mediterranean sublittoral environment.

The bathymetric distribution of the species and the marked summer stratification of Mediterranean Sea water column forces shallow and deep growing plants to adapt to contrasting environmental conditions. Indeed, shallow and deep portions of *P. oceanica* meadows can harbor distinct genotypes (Migliaccio et al., 2005) and show different physiological strategies and gene expression patterns, as assessed in natural conditions (Dattolo et al., 2014), as result of an adaptive divergence toward the light environment experienced at the two depths (Dattolo et al., 2017). This environmental gradient offers an invaluable opportunity to explore potential acclimation/adaptation mechanisms to thermal stress in seagrasses at a small spatial scale. Within the same population, shallow growing plants experience warmer and more fluctuant temperatures than deep ones. Deep meadow portions of the meadow can experience high temperature during extreme thermal events, although for very short periods. Recent findings indicate that genotypes from shallow areas have higher physiological heat acclimation capacities than deep genotypes of the same meadow, living in a colder and more stable thermal environment (Marín-Guirao et al., 2016). Following this findings, tolerant (shallow) and sensitive (deep) genotypes of the species, were collected from above and below the summer thermocline, in a meadow where genetic disjunction between the two depths was previously reported (Dattolo et al., 2017). Genotypes were exposed for several days to an intense warming event in a mesocosms system and photophysiological and transcriptome-wide gene expression analyses were performed after the transient exposure to heat and after a subsequent recovery period. By comparative transcriptomics analysis, we explored genotype's responses to identify mechanisms involved in the tolerance and acclimation of *P. oceanica* to short-term heat events and to provide understanding about seagrasses capacities for plastic and evolved responses to ocean warming. Besides a comprehensive analysis of the transcriptomic profiles of experimental plants, we dig into targeted pathways that are key in acquired thermotolerance in plants (e.g., heat shock proteins and factors, antioxidants, epigenetics) as well as into key physiological processes with known contrasting heat tolerances in the studied species (e.g., photosynthesis and respiration). The objective is to identify transcriptomic traits associated to higher thermal tolerance in seagrasses as well as the potential mechanisms behind the thermal resilience of key physiological processes in the species.

## Materials and methods

### Plant sampling, genotyping, and experimental design

On October 2014, large *P. oceanica* plant fragments bearing a high number of connected shoots were collected by divers at 5 m (shallow) and 25 m (deep) depth within a continuous meadow off the south-eastern coast of Spain (Isla Grosa; 37° 43′ 40″N / 0° 41′ 48″W). Sea water temperature at the sampled meadow annually ranges between 12.94 ± 0.45°C and 28.03 ± 0.93°C with an average temperature for the summer months of July and August of 26.42 ± 0.64°C (Period 2010–2016; Ruiz et al., 2015). Within 2 h of collection, three shallow and three deep plant fragments were split in two portions of similar size and number of shoots (i.e., 20–25 and 14–17 shoots for shallow and deep fragments, respectively) and transplanted in individual tanks (120 L). Genotyping was carried out following the methods described by Tomasello et al. (2009), to ensure that these fragments represent different genotypes. Briefly, a leaf segment of about 2 cm was collected from each plant fragment, cleaned of epiphytes with a razor blade and dried in silica gel. Genomic DNA was manually extracted following the standard protocol of the nucleo-spin plant II kit (Macherey-Nagel). Individual multilocus genotypes were assessed by a total of 12 microsatellites and manually compared for assessing identity (Table S1).

Plants were acclimated under their respective environmental conditions as determined in the field at the moment of sampling. Temperature was the same at the two depths, i.e., 24 ± 0.2°C, salinity was 37.5 ± 0.2 ppt and irradiance was 7.2 ± 0.6 and 1.9 ± 0.3 mol photons m^−2^ day^−1^, for shallow and deep plants, respectively. The photoperiod was 8/16 h. After 10 days of acclimation in the experimental tanks, harboring one of the two plant fragments of each genotype, water temperature was progressively increased to 32°C at a rate 0.5°C h^−1^. The exposure lasted 5 days after which temperature was returned to control levels to allow plants to recover from heat for another 5 days. Plant responses were determined in each tank at the end of both the exposure and the recovery periods. For a more complete description of the experimental system, see Marín-Guirao et al. (2016).

### PSII photochemical efficiency

A diving-PAM fluorometer (Walz, Germany) was used to evaluate the maximum quantum yield of PSII (F_v_/F_m_), a measurement of the photosynthetic efficiency of PSII. For each sample, F_v_/F_m_ was calculated by determining the minimum fluorescence of whole-night dark-adapted leaves (F_0_) and the maximum fluorescence (F_m_) when all PSII reaction centers were reduced by a saturation pulse of white light [F_v_/F_m_ = (F_m_–F_0_)/F_m_]. Measurements were conducted at the end of the exposure and recovery periods on three shoots per tank (i.e., plant fragment) and then averaged to be used as individual replicates (*n* = 3). Data obtained at each time point were analyzed with factorial ANOVA to explore the effects of the heat shock on PSII functionality, and subsequently subjected to *post-hoc* Student-Newman-Keuls test, when significant differences were found for the factors (*p* < 0.05).

### RNA extraction and MRNA sequencing

A 7 cm leaf segment from 20 cm above the ligule of the first mature leaf was collected (14:00 h) at the end of the exposure and recovery periods from one randomly selected shoot per tank. Samples were immediately cleaned of epiphytes, dipped into RNA-later and stored at −80°C until total RNA extraction, that was performed using the Aurum Total RNA Kit (Bio-Rad). The quality and quantity of the extractions were assessed by using Nanodrop (Thermo Fisher Scientific) and a 2100 BioAnalyzer (Agilent). Twenty four libraries (2 depths × 2 treatments × 3 replicates × 2 time points) were constructed with TruSeq Stranded mRNA Library Prep Kit (Illumina) and sequenced with a Hiseq1000 platform (Illumina) to generate a total of 776,971,548 paired-end reads of 101 bp in average (>30 million reads per library; see Table AS1 in Appendix S1).

### *De novo* transcriptome assembly

Quality of raw Illumina sequence data was assessed using FASTQC (v.11.3) software (Andrews, 2010). Raw reads were subjected to a cleaning procedure using Trimmomatic v2.33 (Bolger et al., 2014) and possible post-sequencing ribosomal RNA contamination were eliminated through the local sequence alignment tool SortMeRna v.2.0 (Kopylova et al., 2012) with an *E*-value cut-off of 1E-20. Using Trinity v.2.0.6 (Grabherr et al., 2011) with *in-silico* read normalization and default parameters, HQ cleaned reads were assembled into a transcriptome as no reference genome is available for *P. oceanica*. To further assess the quality of the assembly, reads from each library were mapped back to the assembly using Bowtie v.1.1.1 (Langmead et al., 2009). The assembled transcripts with length ≥200 bp were used as the sequence dataset of reference transcripts.

### Functional annotation

Sequence similarity searches of the Swiss-Prot and NCBI's non-redundant (nr) databases were conducted with BLASTX program (Altschul et al., 1997) with a cutoff *E*-value of 1E-6. Assembled transcripts were also compared against NCBI's partially non-redundant nucleotide sequences databases using BLASTN (*E*-value < 1E-6). Subsequently, with an integrated use of the Blast2GO v.3 (Conesa et al., 2005) we retrieved Gene Ontology (GO) terms (Ashburner et al., 2000) at an *E*-value threshold of 1E-6 for transcripts with a positive BLAST hit and additional functional information based on protein domains and motif information through the InterProScan function available in Blast2GO with default settings (Zdobnov and Apweiler, 2001). We also performed enzyme code (EC) annotation for the assembled transcripts and retrieved KEGG maps for the metabolic pathways in which they participate. In situations in which a given gene contained multiple isoforms, the longest one was defined as the gene functional annotation and used in the following analysis.

### Gene expression and go enrichment analyses

Gene expression analyses were performed using a filtered transcriptome as a reference in order to maintain transcripts that were more strongly supported by the reads. Trinity wrapper run_RSEM.pl (Haas et al., 2013) was used to align reads to the assembled contigs and to select for transcripts that accounted for at least 1% of the per-component (IsoPct) expression and that met a transcript per million (TPM) cutoff of 1. Illumina reads from each biological replicate were aligned separately to the filtered transcriptome using Bowtie v.1.1.1 (Langmead et al., 2009) and expression of each gene was quantified using the Expectation-Maximization method (RSEM; Li and Dewey, 2011). Differentially expressed genes were determined using the edgeR package (Robinson et al., 2010). Firstly, we selected a stringent cut-off of four-fold (FDR < 0.001; herein after referred as strongly differentially expressed genes, s-DEGs) to explore overall responses and to examine differences in gene expression in the following pair wise comparisons: (i) all controls vs. all heated plants, to explore the general heat stress response in *P. oceanica* and; (ii) shallow controls vs. shallow heated plants and deep controls vs. deep heated plants, to study the response to heat of shallow and deep plants, respectively. All the comparisons were performed after both the exposure and the recovery periods. Secondly, DE analysis was broadened to a level of two-fold change (FDR < 0.01, herein after referred as differentially expressed genes, DEGs) and shallow and deep plants were compared with their controls (ii of the previous analysis) only for specific key physiological processes (i.e., photosynthesis and respiration) involved in contrasting heat tolerances among shallow and deep *P. oceanica* plants (Marín-Guirao et al., 2016). Epigenetic processes, heat shock factors (HSFs), HSPs, and antioxidants production were also considered, due to their important implications in plant thermotolerance (Wahid et al., 2007; Bita and Gerats, 2013).

Principal component analysis (PCA) was conducted using the “prcomp” function within R environment v. 3.2.2 (R Core Team, 2015) and scatter plot of PCA results was generated using the ggplot2 package (Wickham, 2010).

GO enrichment analyses of DEGs were performed using Blast2GO with a threshold FDR-corrected *P*-value from Fisher's exact test, 0.05. Summarization of GO terms based on their semantic similarities was performed on the REVIGO web service (http://revigo.irb.hr/; Supek et al., 2011) and their representation on a 2D semantic space was generated using the ggplot2 package in R.

To validate results of our sequencing analyses (mRNA-seq), the expression of 12 randomly selected genes was assessed by Reverse Transcription—quantitative Polymerase Chain Reaction (RT-qPCR). We used the same shallow- and deep-RNA samples from the end of the heat exposure period that were utilized for transcriptome sequencing, and followed the methods described in Mazzuca et al. (2013). Complementary DNA was synthetized with the iScriptTM cDNA Synthesis Kit (Bio-Rad). RT-qPCR was performed in triplicate in a Viia7 Real Time PCR System (Applied Biosystem) using Fast SYBR® Green Master Mix (Thermo Fisher Scientific) and analyzed by the 2^−Δ*ΔCt*^ method, using L23 and NTUBC as reference controls (Marín-Guirao et al., 2016). All primer sequences used for RT-qPCR are listed in Table S2.

## Results

### Effects of short-term heat stress on PSII photochemistry

**Figure 1:**
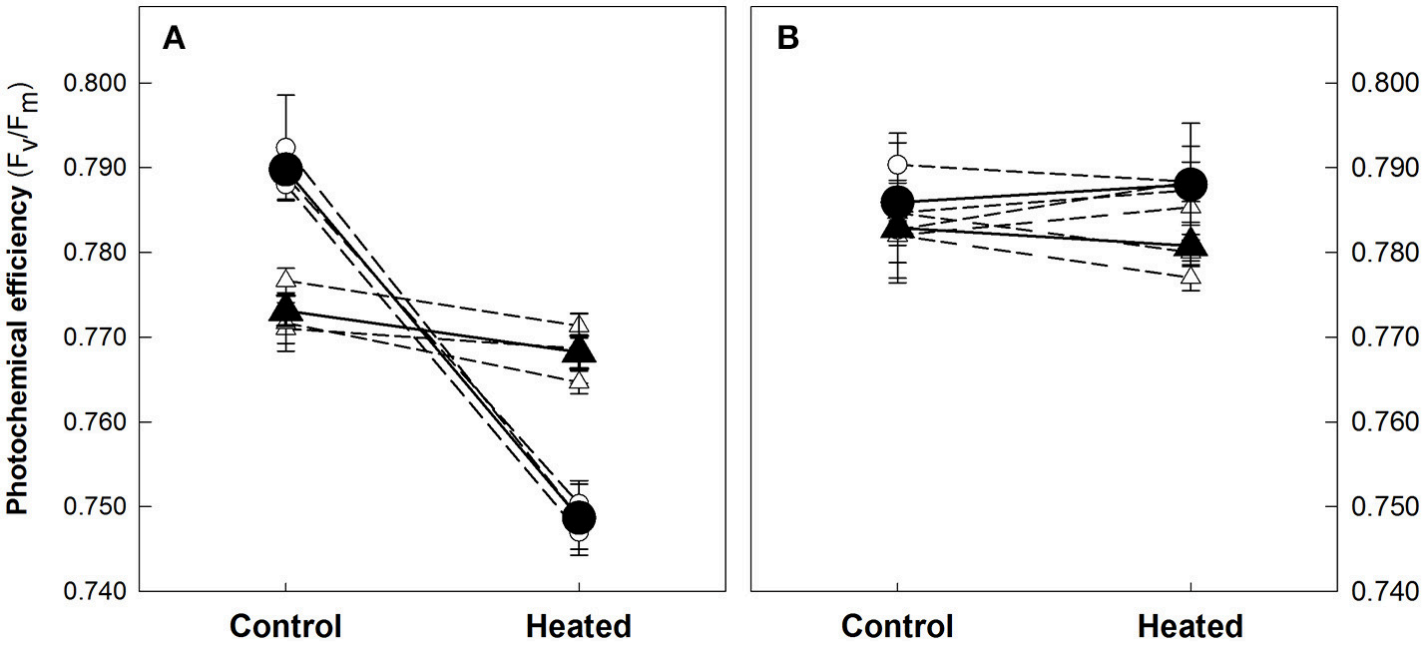
Maximum PSII photochemical efficiency (F_v_/F_m_) of shallow and deep *Posidonia oceanica* plants from the control and heat treatments at the end of the heat exposure **(A)** and recovery **(B)** periods. Averaged F_v_/F_m_ values (± standard error) of the three shoots measured in each individual genotype (empty symbols) and the average value of the three replicate genotypes (solid symbols) are represented. Plant's depth is represented with different symbols as showed in the legend.

Exposure of shallow plants for 5 days to the experimental temperature of 32°C did not cause any significant alteration on the photochemical efficiency (F_v_/F_m_). Deep plants, instead, showed a significant reduction (*p* < 0.001) of the F_v_/F_m_ ratio, from an average of 0.790 (± 0.001, SE) in controls to 0.749 (± 0.001) in heated plants (Figure 1). This reduction was due to a significant 35% increase in F_0_, indicating that the applied stress caused PSII inactivation (Brestic and Zivcak, 2013). Photochemical effects disappeared after 5 days of recovery when controls and heated plants from both depths showed similar photochemical efficiencies, with values that in all cases were above 0.780.

### Transcriptome sequencing and *De novo* assembly

A thorough description of the results derived from the sequencing, *de novo* assembly and annotation of the transcriptome is provided as supplementary information (Appendix S1). In brief, a total of 697,043,338 high quality paired-end reads (89.7% of initial raw reads) were *de novo* assembled resulting in 222,548 putative transcripts (≥200 bp) with an overall size of 390 Mb, an average length of 918 bp and GC percent of 44.5%. On average, 82.7% of the reads mapped back to the assembly indicating that the transcriptome was a reliable reference. Raw sequence reads were deposited in NCBI Short Read Archive under BioProject ID PRJNA353749 (accession number SRP093709).

### General transcriptomic response of *P. oceanica* to heat stress

**Figure 2:**
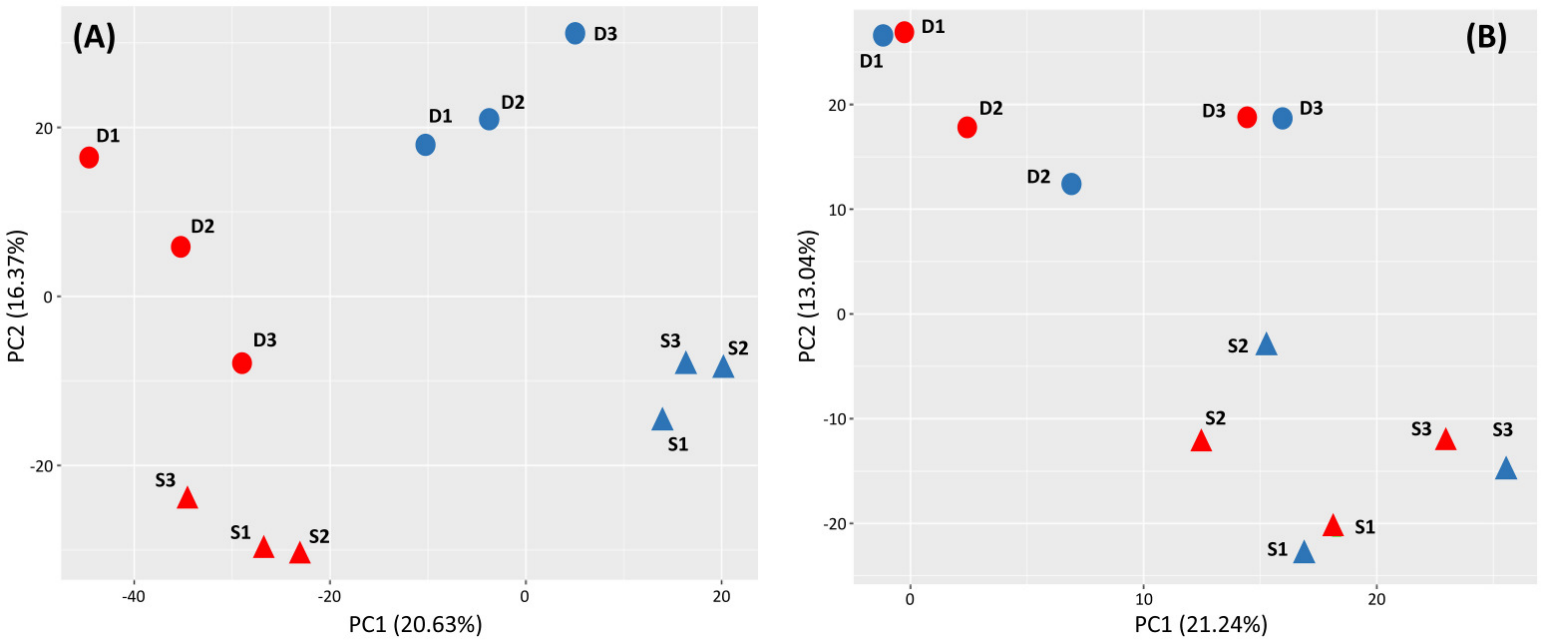
Principal component analyses (PCA) performed with all 72,169 differentially expressed contigs (5% false discovery rate) found in shallow (triangles) and deep (circles) *Posidonia oceanica* plants at the end of the heat exposure **(A)** and recovery **(B)** periods. Heated and control plants are represented in red and blue, respectively. In parenthesis, the proportion of variance explained by principal components 1 and 2 is represented.

The patterns of gene expression following heat stress was consistent among replicates (i.e., genotypes; Figure 2) and hierarchical cluster analysis of s-DEGs clearly revealed different patterns of expression between heated and control plants (pooled across depths; Figure S1).

**Figure 3:**
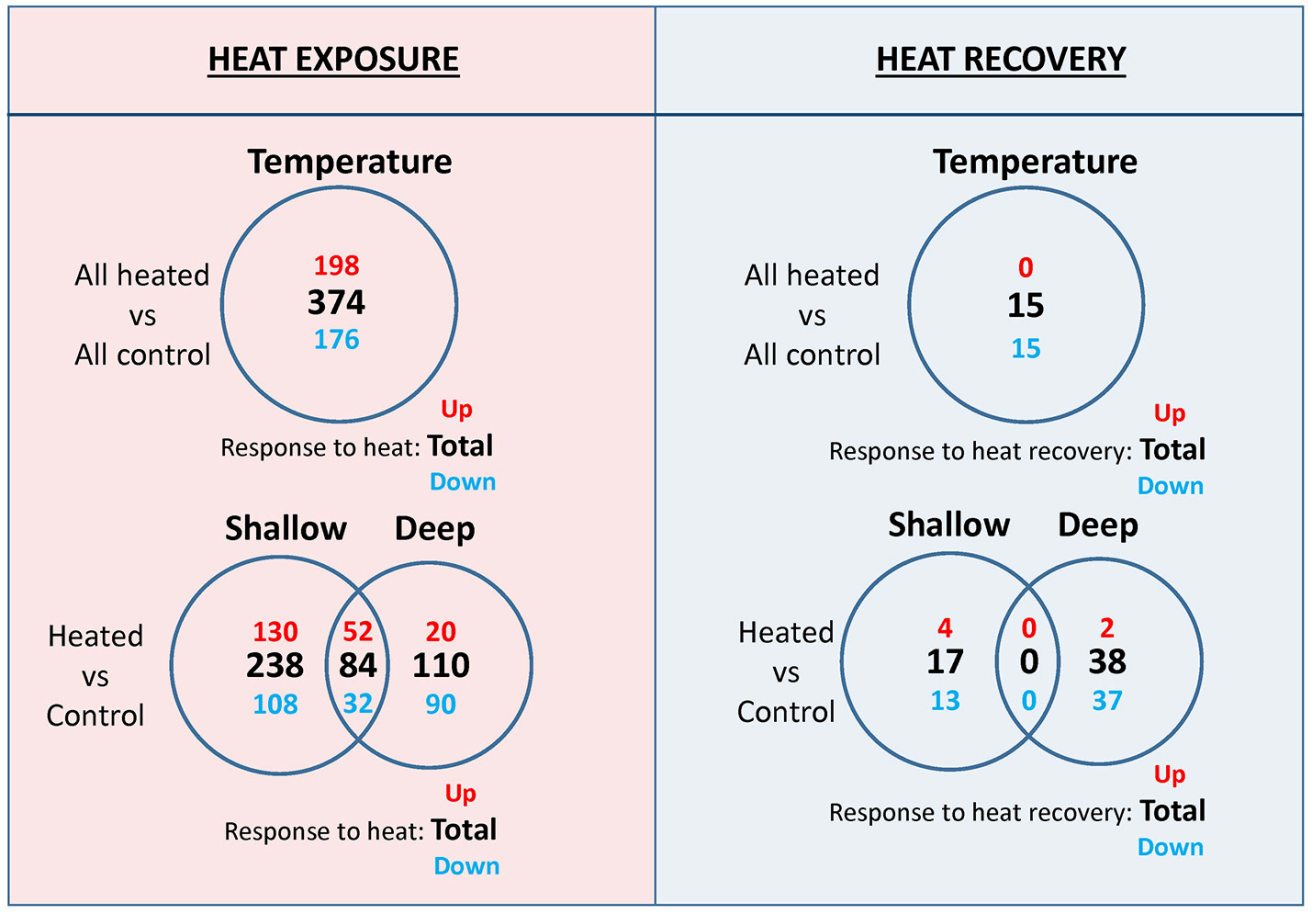
Venn diagram showing the number of strongly differentially expressed genes (s-DEGs; >four-fold change; false discovery rate<0.001) based on temperature, depth, within-depth heat response, and within-treatment depth differences at the end of the heat exposure (left panel) and at the end of the heat recovery (right panel) periods. Bold numbers denote totals, while red and blue numbers indicate, respectively, up-regulated and down-regulated or higher expression in shallow and in deep plants, as indicated in the figure.

The comparison among all heated and all control plants showed the largest number of s-DEGs (#374, Figure 3) among all the other comparisons (i.e., shallow controls vs. shallow heated and deep controls vs. deep heated), with 198 genes up-regulated with an average fold change of 6.8 (FC range, 4–265). The most highly up-regulated genes were an adenylate cyclase and a photosystem II 10 kDa chloroplastic protein (psbR) associated with the oxygen-evolving complex of PSII (Data S1A). Over-expressed biological functions of up-regulated genes (Fisher test, FDR < 0.05) included among others: (1) responses to stimulus and stress (including heat); (2) DNA and RNA processes (DNA repair, replication, codification, transcription and methylation; gene silencing by RNA, regulation of RNA process); (3) regulation of cellular biosynthetic and metabolic processes and of cell growth and division; and (4) cell wall organization (Data S2A). Down-regulated genes (#176, Figure 3) for the same comparison were enriched in 41 over-represented biological processes, with the glycerolipid biosynthetic process being the most representative one (*p* = 1.05E-08; Data S2B). After a recovery period of 5 days, the comparison “all heated vs. all controls” showed that differences in gene expression almost disappeared since only 15 s-DEGs (all down-regulated, Figure 3) were detected. Genes involved in key processes that could potentially compromise the long-term performance of heated plants after stress cessation were not identified within this set of genes (Data S1B). The most highly down-regulated genes encode for key enzymes of the jasmonic acid biosynthesis pathway (lipoxygenase 5 and allene oxide synthase), which are tightly involved in plant stress responses (Yang et al., 2011). Two genes encoding HSPs and that were previously upregulated during the heat exposure were also identified in this set of genes.

### Depth-specific transcriptomic response

In the PCA plot, obtained using normalized expression values, heated and control plants showed the strongest differentiation and were clearly separated along the first axis (PC1 = 20.6% of the total variance), while PC2 axis (16.4% of total variance) differentiated shallow from deep plants irrespective of the thermal treatment. The PCA conducted after recovery still differentiated shallow from deep plants (PC2 = 13.04% of the total variance) whereas it grouped all heated genotypes from both depths with their own controls, indicating their successful recovery (Figure 2). Also in the heatmap derived from hierarchical cluster of s-DEGs, the two main clusters of heated and control plants contained two sub-clusters for shallow and deep plant replicates, respectively (Figure S1). After the recovery period, however, transcriptomic differences due to heat disappeared and all shallow and deep replicates were clustered together irrespective of whether they were previously heated or not.

### Shared transcriptomic response to heat stress between depths

There were 84 genes that commonly responded to heat stress in shallow (shallow heated vs. shallow controls) and deep (deep heated vs. deep controls) plants, 52 up-regulated and 32 down-regulated (Figure 3). Common up-regulated genes during heat exposure (Table 1) were involved in protein folding (11 genes), oxidation-reduction processes (3 genes), ubiquination and proteolysis (3 genes), membrane and cell wall modification (2 genes), carbohydrate metabolic processes (2 genes), regulation of cell cycle (1 gene), cyclic phosphorylation (1 gene) and in the heat shock mediated-signaling pathway (1 gene, i.e., serine threonine-protein phosphatase 7; Liu et al., 2007). These common up-regulated genes showed, however, differences in their expression strength and constitutive levels (i.e., normalized contig reads in controls) between shallow and deep plants (Table 1). While the most highly up-regulated genes in shallow plants were involved in protein folding and encode for HSPs, those of deep plants were related to ubiquination (u-box domain-containing protein 12-like, PUB12) and proteolysis (tripeptidyl-peptidase 2, TPP2). Besides a few HSPs, the largest difference in the responsiveness to heat between shallow and deep plants corresponded to chloroplastic polyphenol oxidase A1, which also has higher constitutive level of expression (Table 1) and is likely involved in protecting photosynthesis functioning, increasing stress tolerance in plants (Boeckx et al., 2015). Moreover, in shallow plants 74% of all common genes showed stronger heat response compared to deep plants and 68% showed higher constitutive expression (Table 1).

**Table 1:** List of annotated s-DEGs (>4 FC, FDR < 0.001) commonly up-regulated in shallow (S) and deep (D) *P. oceanica* plants under heat stress.

		**Responsiveness to heat**			**Constitutive expression levels**		
**Contig ID**	**Description**	**S heated (FC)**	**D heated (FC)**	**S/D (FC ratio)**	**S controls**	**D controls**	**S/D (controls ratio)**
TR12261|c0_g1	bag family molecular chaperone regulator 6-like	26.5^*^	13.9^*^	1.9	1.57	1.99	0.79
TR28282|c0_g1	BI1	12.6	10.0	1.3	2.70	1.15	2.35
TR16275|c6_g1	cell wall transcription factor ACE2-like	4.7	6.9	0.7	1.22	0.85	1.44
TR44736|c0_g1	Chaperone-domain superfamily isoform 1	4.6	6.8	0.7	1.87	1.32	1.42
TR14486|c4_g3	conserved hypothetical protein	8.3	7.3	1.2	105.00	70.93	1.48
TR22140|c3_g6	disease resistance RPP13 1	**34.5**	**21.8**	1.6	0.35	0.40	0.87
TR44650|c0_g1	DNA topoisomerase 3-alpha	4.6	4.3	1.1	1.44	2.99	0.48
TR44578|c1_g2	dnaj protein homolog	12.6	5.2	2.4	65.24	43.27	1.51
TR35505|c0_g1	formate dehydrogenase, mitocondrial	4.8	6.2	0.8	1.33	2.05	0.65
TR15373|c0_g1	galactinol synthase 2-like	23.6	9.7	2.4	1.11	0.51	2.19
TR22140|c3_g5	potassium channel KAT3	8.0	7.6	1.1	0.82	1.50	0.55
TR44569|c0_g1	hypothetical protein VIGAN_UM125500, partial	21.9	9.2	2.4	5.81	8.91	0.65
TR4038|c2_g1	Chain A, N-terminal Domain Of Heat Shock 90 From Oryza Sativa	27.8^*^	10.1^*^	2.8	48.66	43.53	1.12
TR4038|c3_g1	heat shock protein 83	34.6^*^	9.1	3.8	57.19	43.80	1.31
TR37115|c0_g1	hemiasterlin resistant 1-like	16.1	8.7	1.8	4.16	4.06	1.03
TR14486|c4_g2	hypothetical protein B456_003G156400	8.4	7.9	1.1	19.96	8.67	2.30
TR14486|c4_g1	hypothetical protein LR48_Vigan03g091700	14.3	6.9	2.1	15.58	13.37	1.17
TR21156|c1_g2	serine threonine-phosphatase 7 long form homolog	4.5	6.2	0.7	5.24	4.89	1.07
TR32305|c4_g1	kDa class I heat shock-like	32.0^*^	12.0^*^	2.7	64.96	52.47	1.24
TR32305|c4_g2	kDa class I heat shock-like	34.6^*^	9.7	3.6	46.90	52.70	0.89
TR34507|c1_g1	small heat shock	26.5^*^	9.2	2.9	28.51	19.66	1.45
TR34507|c2_g1	kDa class I heat shock protein	44.8^*^	9.1	4.9	26.62	32.62	0.82
TR25079|c5_g1	lysosomal beta glucosidase-like	5.0	7.1	0.7	1.10	0.53	2.06
TR32158|c0_g1	Multi-bridging factor 1c	14.3	**10.6**	1.4	6.99	4.74	1.48
TR3461|c2_g1	NRT1 PTR FAMILY-like	4.9	9.2	0.5	0.55	0.66	0.84
TR324|c2_g1	Polyphenol oxidase A1, chloroplastic	**31.5**	8.9	3.5	13.26	4.28	3.10
TR1353|c0_g1	PREDICTED: uncharacterized protein LOC103710593 isoform X1	4.7	4.0	1.2	14.43	11.07	1.30
TR24633|c1_g1	PREDICTED: uncharacterized protein LOC104585875	8.0	7.2	1.1	1.96	0.93	2.11
TR31935|c1_g1	E3 ubiquitin-ligase SINAT2-like	4.8	4.8	1.0	37.13	35.66	1.04
TR43397|c4_g4	serine threonine-protein phosphatase 7 long form homolog	23.7	**16.1**	1.5	0.70	0.09	7.62
TR28865|c4_g3	kDa heat shock, mitochondrial	28.6^*^	10.4^*^	2.7	4.31	5.44	0.79
TR31595|c0_g2	Thioredoxin H-type	15.4	7.6	2.0	3.36	2.26	1.49
TR10497|c0_g6	tripeptidyl-peptidase 2	20.6	**25.3**	0.8	0.12	0.45	0.27
TR24126|c0_g1	u-box domain-containing protein 12-like	12.0	**52.1**	0.2	0.30	0.07	4.15
	**Higher in shallow (% of genes)**			**73.5**			**67.6**

For each gene the fold expression change and the constitutive expression level (normalized contig reads x1,000) as well as their respective ratios are given. The 10 most expressed common genes in shallow and deep plants exposed to heat are shown in bold, among which heat shock proteins and molecular chaperons are highlighted with asterisks (^*^).

### Difference in the response to heat stress between depths

Besides the above differences in the set of genes that commonly react to heat, *P. oceanica* plants from different depths also showed other important differences. In the comparison with their own controls (“heated vs. control”), shallow heated plants showed 40% more s-DEGs than deep plants (322 vs. 194), and the proportion was 60% higher for up-regulated genes (182 vs. 72) and 13% higher for down-regulated ones (140 vs. 122) (Figure 3). These differences were even higher when considering genes that react to heat exclusively in plants of one of the two depths, mainly due to the much higher number of unique up-regulated genes in shallow plants (130 vs. 20) since the number of unique down-regulated ones was almost similar (108 vs. 90).

**Figure 4:**
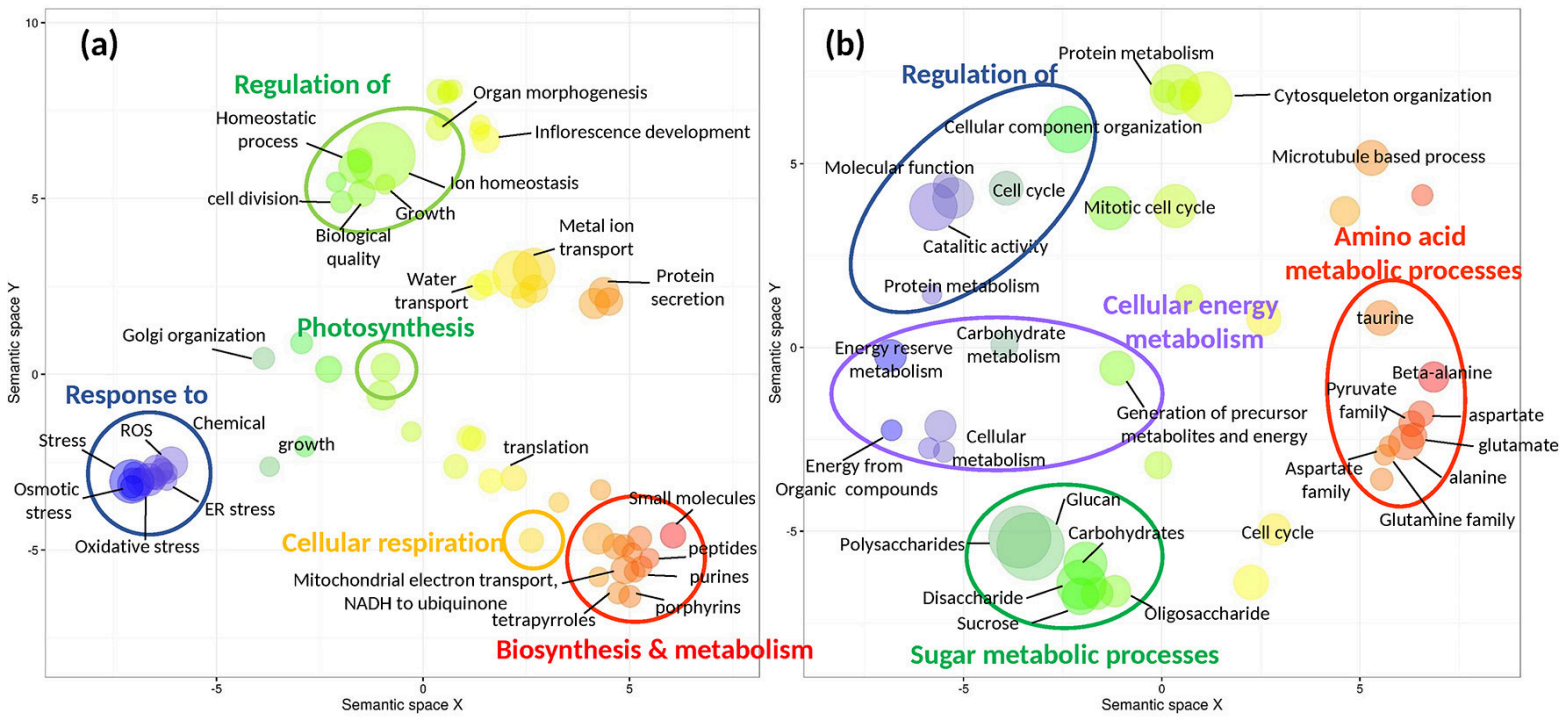
Functional enrichment (Fisher test, false discovery rate<0.001) of heat-responsive shallow-unique **(a)** and deep-unique **(b)** s-DEGs (>4 FC, FDR < 0.001). Gene ontology (GO) terms of biological processes are represented by bubbles and plotted according to semantic similarities to other GO terms. Bubble size is proportional to log_10_ *p*-values of the GO term, while color indicates semantic similarities. The two-dimensional semantic space was generated by the REVIGO web service with all GO terms found in the enrichment analyses. REVIGO's tables containing all gene functional categories represented in **(a)** and **(b)** are given as supplementary information (Data S3A and S3B).

This set of shallow-unique up-regulated genes was enriched (Fisher test, FDR < 0.05) in biological processes related, among others, to: (1) response to stress (oxidative stress, reactive oxygen species (ROS), endoplasmic reticulum (ER) stress); (2) biosynthesis and metabolism (tetrapyrroles, porphyrins, peptides); (3) regulation of homeostatic processes and growth; (4) photosynthesis and; (5) cellular respiration and respiratory electron transport chain (ETC; Figure 4A; Data S2C). The enrichment analysis of deep-unique up-regulated genes showed over-represented biological functions related to (1) amino acid and (2) sugar metabolic processes; (3) cellular energy metabolisms and (4) regulation of catalytic activity and protein metabolism, among others (Figure 4B; Data S2D).

**Figure 5:**
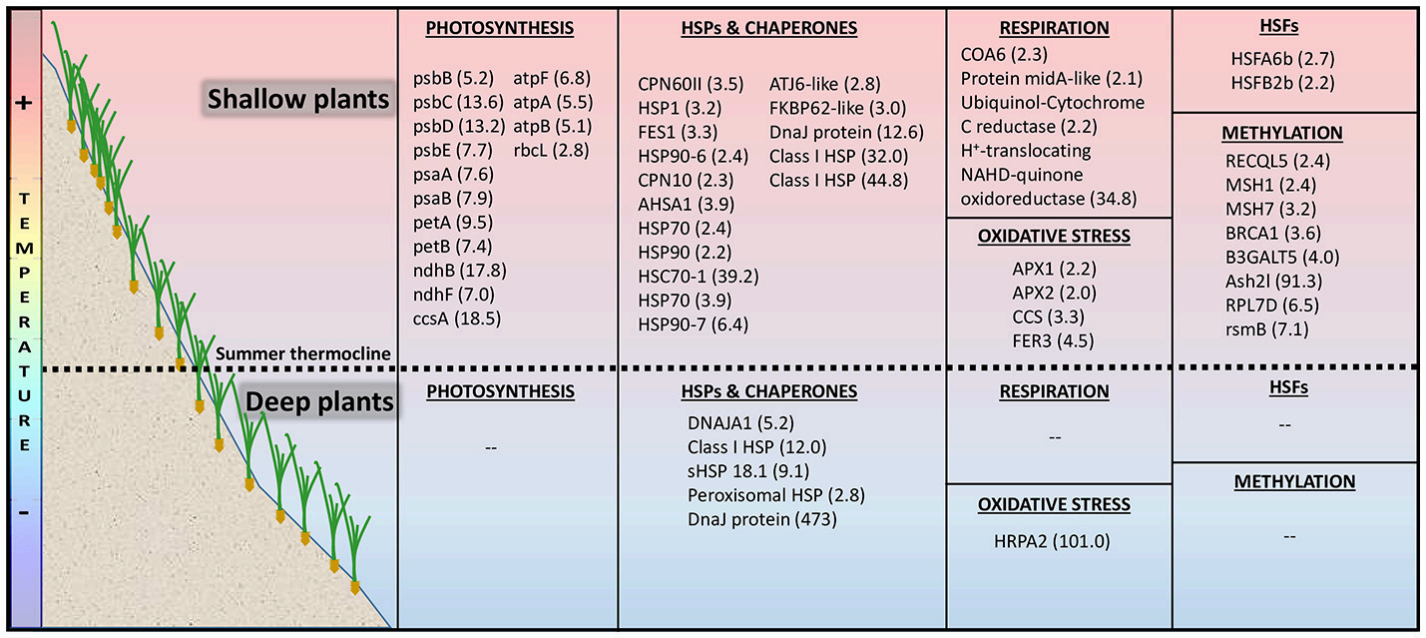
Depth-specific heat transcriptomic response of *Posidonia oceanica* plants living above (shallow 5 m) and below (deep 25 m) the summer thermocline after being exposed to an increased temperature of 32°C during 5 days. The table shows differentially expressed genes (s-DEGs and DEGs) exclusively up-regulated in each of the two depths and their fold change in brackets.

Strongly up-regulated unique-shallow genes (s-DEGs, FC > 4, FDR < 0.001) included a rich set of photosynthesis-related genes (Figure 5). They encode several proteins of the PSII complex such as the reaction center protein D2 (psbD), the light-harvesting proteins CP43 (psbC) and CP47 (psbB) and the cytochrome b559 alpha (psbE, essential for PSII assembly; Burda et al., 2003), as well as the two main subunits of PSI (psaA and psaB; Figure 5; Data S1Ca). This set also included genes that encode proteins of the photosynthetic ETC, such as the NADH-plastoquinone oxidoreductase subunits 2 and 5 (ndhB and ndhF), the cytochrome C heme attachment (ccsA) and two subunits of the cytochrome b6-f complex (petA and petB), together with several components of the ATP synthase complex (atpA, atpB, and atpF). Shallow plants also exclusively activated a strong transcription of a proton-translocating NADH-quinone oxidoreductase of the mitochondrial respiratory ETC (complex I). Other s-DEGs were HSPs and molecular chaperons involved in protein folding such as hsp70 nucleotide exchange factor fes1, heat shock 70, heat shock cognate 70-1 and endoplasmin homologHSP90-7 (Figure 5; Data S1Cb).

Less strongly up-regulated unique-shallow genes (DEGs, >2 FC, FDR < 0.01; Data S1Cb) also comprised the ribulose-1,5-bisphosphate carboxylase/oxygenase large subunit (rbcL), a broad group of HSPs and molecular chaperons and two heat shock transcriptional factors (Figure 5). Other components of the mitochondrial respiratory ETC were also uniquely up-regulated in shallow heated plants and included the amidA-like protein required for complex I function (Carilla-Latorre et al., 2010), the ubiquinol-cytochrome-c reductase (complex III) and the cytochrome C oxidase assembly factor 6 required for the efficient formation of the respiratory chain complex IV (Stroud et al., 2015). This only-shallow heat-activated set of genes also comprised few genes involved in the defense against oxidative stress such as L-ascorbate peroxidase 1 and 2 (APX1 and APX2) and a copper chaperone for the activation of superoxide dismutase (CCS), which is involved in the regulation of the heat stress response and plant thermotolerance (Guan et al., 2013).

Contrarily, deep heated plants did not exclusively activate genes involved in photosynthesis or in respiration and just a few HSPs and molecular chaperones (Figure 5; Data S1Ea and S1Eb). HSFs were neither included in the set of only deep up-regulated genes. The most highly expressed genes were not all included in the functional categories illustrated in Figure 5. Among the others, a suppressor of gene silencing (436 FC) and a gene implicated in hydrogen peroxide catabolism (Peroxidase A2–HRPA2, 101 FC) were present. Other deep up-regulated genes were related to proteolysis (subtilisin-like protease, 14 FC) and ubiquination (5.5 FC).

Both shallow and deep plants expressed TOP3, a gene related to DNA or histone methylation. Besides that one, shallow plants up-regulated 8 additional genes related to methylation (Figure 5) and down-regulated a gene involved in demethylation (Lysine-specific demethylase 5B, not in Figure 5).

After being allowed to recover from heat stress, shallow and deep plants showed respectively 17 and 38 DE genes in respect to their controls (Figure 3; Data S1D and S1F), most of them being down-regulated (13 and 37, respectively) and including HSPs previously up-regulated during the heat exposure. At this time, there were no shared genes among plants from the two depths.

### Validation of RNAseq results

All tested genes assessed through RT-qPCR successfully validated RNA-seq results in shallow and deep genotypes, although showing minor differences in the magnitude of expression (i.e., fold changes; Figure S2). Results from both analyses were significantly correlated both in shallow (*N* = 12, *r* = 0.89, *p* < 0.001) and deep (*N* = 12, *r* = 0.90, *p* < 0.001) genotypes.

## Discussion

The results of our transcriptomic analysis show that the iconic seagrass *P. oceanica* is able to overcome and recover from several days of severe heat. Species tolerance and resilience to heat stress is supported by the lack of transcriptomic evidences of lethal and permanent injury and by the complete photophysiological recovery. The activation of overall metabolic reprogramming seems to be the basis of the species' ability to withstand short-term heat stress. Some of the observed responses were aimed to restore the cell wall rigidity for ensuring the cell architecture and growth (Houston et al., 2016). They included the induction of the genes CEL3 (endoglucanase 9) and POK2 (phragmoplast orienting kinesin 2-like), required for cell wall organization (Robertson et al., 1997; Müller et al., 2006), and the reduction in the synthesis of glycerolipids, which is considered advantageous for the long-term acclimation of plants under increased temperatures (Higashi et al., 2015). Similar responses have been reported in the seagrass species *Z. marina*, where the enhanced expression of genes involved in cell-wall fortification buffer heat-stress and increase thermotolerance (Franssen et al., 2014; Jueterbock et al., 2016). The plants also show common mechanisms of recovery after the heat stress. Plants mostly shutdown processes involved in the thermal tolerance that were activated during exposure, as for instance HSPs. Moreover, the strong down-regulation of the genes LOX5 and AOS is possibly reflecting the de-activation of the stress signaling orchestrated by jasmonic acid (Ahmad et al., 2016). Our results also show important differences in the transcriptomic profiles of heated plants from different depths that reflect their different physiological tolerance and acclimation capacity to transitory warming and that might differentially affect plant survival under longer warming events. The observed transcriptomic differences were mainly based on: (i) the number of genes that exclusively react in plants of different depths and the physiological functions associated to them, (ii) the expression strength of the genes that commonly respond at both depths, and (iii) the constitutive levels of the commonly responsive genes.

### Differential heat sensitivity as resulting from the depth-specific transcriptomic response to heat

**Figure 6:**
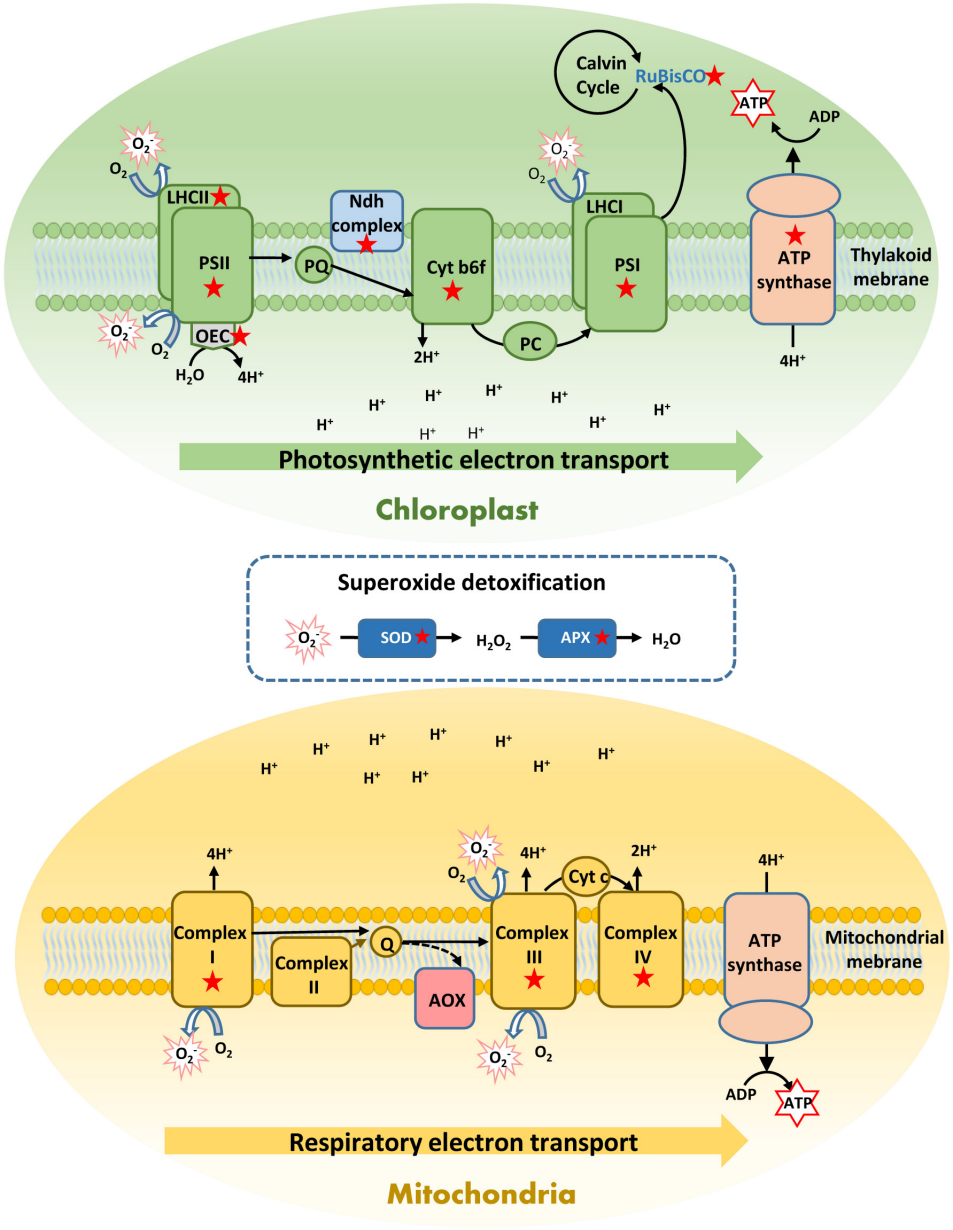
Schematic representation of a respiratory and photosynthetic electron transport chains (ETC) showing the components in which heat-induced genes in shallow *Posidonia oceanica* plants are involved (labeled with a red star). Heating induced over-expression of a rich set of photosynthetic genes to improve the turnover of functional and structural proteins and maintain an efficient photosythetic performance and ATP production. In addition, heat stress uniquely induced in shallow plants over-expression of respiratory genes related to the mitochondrial ETC complexes I, III, and IV, which reflect the activation of the cytochrome pathway for respiratory ATP production. Although this pathway is more energy-efficient than the alternative oxidase pathway (AOX), it is also the main producer of mitochondrial ROS, especially the superoxide radical (${\text{O}}_{2}^{-}$). To protect against oxidative damage these shallow plants activated the ROS-scavenging enzymes superoxide dismutase (SOD) and ascorbate peroxidase (APX). These exclusive responses of shallow plants conferred them higher heat tolerance respect to deep plants, and enabled them for an efficient energy production to cover the energetic cost associate to heat stress. A complete list of these genes with their corresponding fold change is shown in Figure 5 and in Data S1.

In shallow plants, heat activated a greater number of genes reflecting the induction of a more complete heat response compared to deep plants, especially related to key physiological processes, such as photosynthesis and respiration. This also matches with the higher photosynthetic stability here observed in shallow-heated plants and with their higher respiratory acclimation previously reported (Marín-Guirao et al., 2016). Hence, gene activation seems to be more important than gene suppression in *P. oceanica* thermo-tolerance, as also supported by the presence of many shallow-unique up-regulated genes encoding proteins involved in acquired thermo-tolerance (e.g., HSPs, antioxidants). Shallow *P. oceanica* genotypes, in fact, showed transcriptomic evidence of suffering lower heat-induced physiological injury compared to deep genotypes. These were able to primarily up-regulate HSPs and molecular chaperons, investing in refolding reversibly denaturated proteins (Evans and Hofmann, 2012; Somero, 2012). Only shallow plants showed an intense transcription activity of a wide set of photosynthesis-related genes, likely to support a proper photosynthetic functioning and ATP production, in order to cope with the increased energy demand (Sharkey and Zhang, 2010; Liu et al., 2014). These genes were involved in all main phases of the photosynthetic process, from light harvesting to carbon fixation. They encode functional and structural proteins of all components of the thylakoid ETC, including the ATP synthase complex (Figure 6), which are known to be directly impacted by heat stress on terrestrial plants (Bita and Gerats, 2013). This strong induction could favor the turnover of relevant parts of the photosynthetic apparatus to maintain a correct electron flow and to protect thylakoid membranes from heat impairment (Song et al., 2014).

Plants from deep meadow portions, on the contrary, accumulated heat-induced damage on their photosystems reducing their photosynthetic efficiency and impairing the photosynthetic energy production. *P. oceanica* plants from deep meadows' portions sharply increase their respiratory activity during heat stress (Marín-Guirao et al., 2016), a metabolic response that allows to meet the increased demand of energy to support higher rates of biosynthesis and protein turnover (Lambers et al., 2008). The upregulation of ubiquination- and proteolysis-related genes suggests that, after 5 days at 32°C, severe protein damage occurred, which required their removal by proteolytic processes. Indeed, the set of genes uniquely induced in deep plants included genes related to the ubiquitin-mediated protein degradation, which is the principal mechanisms of protein catabolism to facilitate plant proteostasis under stressfull conditions (Kurepa et al., 2009). Deep plants were also functionally enriched in metabolic processes for obtaining extra energy from sugars and amino acids (Figure 4). The use of carbohydrate and amino acid reserves could cause long term metabolic and energetic imbalances, which likely translates into impaired fitness and survival (Alcoverro et al., 2001). Deep-growing *P. oceanica* genotypes are, therefore, more fragile in response to extreme heat events, whose intensity and duration are predicted to increase in the coming decades as a result of climate change (Schar, 2016).

It has been shown that shallow *P. oceanica* plants have higher respiratory heat-acclimation capacity in respect to deep plants, which allow them to achieve respiratory homeostasis after several days of warming (Marín-Guirao et al., 2016). In the present study, shallow plants responded to heat with the induction of genes encoding respiratory enzymes of the mitochondrial ETC (Figure 6). This reflects a preference for respiratory ATP production via the cytochrome pathway, which is more energy-efficient than the alternative pathway favored in stressed plants through the enzyme AOX (Millenaar and Lambers, 2003). An induction of the cytochrome pathway was also shown in trees, where some species are able to maintain respiratory homeostasis along temperature gradients (Searle et al., 2011; Searle and Turnbull, 2011). Nevertheless, one of the main roles of the alternative pathway is to limit the formation of mitochondrial ROS, especially the superoxide radical (${\text{O}}_{2}^{-}$) that is mainly produced in complex III (Vanlerberghe, 2013; Deng et al., 2015). The promotion of the cytochrome pathway would therefore not only increase the energy yield of respiration but also ROS production. The accumulation of transcripts that activate and encode the most effective enzymatic antioxidants involved in scavenging of ${\text{O}}_{2}^{-}$ (Teotia and Singh, 2014), suggests that shallow plants also enhance their antioxidant defense, to protect themselves from an increased oxidative damage (Figure 6).

In synthesis, the combined induction of cytochrome-related respiratory enzymes and antioxidant enzymes should be functional, together with the maintenance of an efficient photosynthetic energy production, to the ability of shallow plants for thermal acclimation of respiration (Panda et al., 2008; Kühn et al., 2015).

### Expression strength and constitutive levels of heat-responsive genes

In addition to a more complete transcriptomic response in term of number of heat-responsive genes, shallow *P. oceanica* plants also showed higher expression levels of most of the commonly activated genes. This response matches the one observed in the seagrass *Z. marina*, where plants from warm environments (i.e., southern populations) had higher thermal resilience and showed higher heat-inducible responses than plants from colder environments (northern populations; Franssen et al., 2014). When compared to deep *P. oceanica* genotypes, those growing at shallower depths also showed higher constitutive expression levels of heat responsive genes. The same pattern was recently observed in natural *P. oceanica* populations, confirming a more complex and complete constitutive response at shallower depth (Dattolo et al., 2013, 2014; Procaccini et al., 2017). Similar differences, but among individuals from contrasting latitudes, have been observed in different marine organisms, including other seagrasses (Franssen et al., 2014), corals (Barshis et al., 2013) and marine snails (Gleason and Burton, 2015), and have been suggested to be the result of an adaptive mechanism (or pre-adaptive defense strategy) that confers higher thermal tolerance to cope with frequent heat stress. The wide bathymetric distribution of *P. oceanica* meadows implies that shallow plants growing above the summer thermocline experience higher thermal stress levels than deep ones, similarly to the contrasting thermal environments experienced by organisms from latitudinal distant populations.

### Epigenetic control of heat responses?

We can speculate that life in a more stressful environment may have speeded up stress adaptive processes through epigenetic mechanisms such as DNA methylation and chromatin remodeling. Stress-induced epigenetic mechanisms are crucial in activating the plants' immediate response to stress, as well as in the establishment of short- and long-term adaptation, due to their important role in regulating the expression of stress-related genes (Liu et al., 2015). Consequently, the exclusive induction of genes involved in DNA and histone methylation in shallow heated plants could support the role of this processes in *P. oceanica*, favoring their efficient short-term heat acclimation through the successful activation and regulation of heat-responsive genes (Correia et al., 2013). Accordingly, only these shallow plants showed the activation of heat shock factors, which are transcriptional activators of heat shock genes (Kotak et al., 2007; von Koskull-Doring et al., 2007) and that mutually progress with epigenetic processes in regulating the abiotic stress responses in plants. These epigenetic modifications can be involved in the evolution of adaptive strategies and speciation (Flatscher et al., 2012; Smith et al., 2016) and potentially also in the genetic disjunction existing between shallow and deep meadow stands in *P. oceanica* (Migliaccio et al., 2005). Epigenetic modifications could also be at the basis of the survival of millenary clones of the species in the face of the environmental changes they have experienced along their evolutionary history (Arnaud-Haond et al., 2012). Indeed, epigenetic variation is thought to be of particular importance to provide ecological and evolutionary adaptation to clonal plants, being the likely explanation to their widespread success and long-term persistence (Dodd and Douhovnikoff, 2016; Latzel et al., 2016). Understanding the role of epigenetic processes in enhancing phenotypic plasticity in *P. oceanica* can be very important to improve our ability in predicting the future of this key species. Plasticity, in fact, provides a buffer against rapid climatic changes and also assists the rapid adaptation of the species to the ongoing climatic change (Merila and Hendry, 2014).

Our experimental procedure allowed us to identify key mechanisms involved the thermal tolerance of *P. oceanica*. However, since we did not simulate a realistic summer heatwave in terms of rates and duration of warming, we cannot conclude that this keystone species is able to overcome the heat stress produced during these natural extreme thermal events. In a global change scenario, in which the Mediterranean Sea is undergoing increases in the duration of extreme warming events, the effect of heat stress on *P. oceanica* meadows could be dramatic (Jordà et al., 2012). *P. oceanica* plants from above and below the summer thermocline have differential resilience to heat and meadow portions from different depths are expected to be differentially impacted by these extreme events. Shallow plants, living in a more unstable thermal environment, optimized phenotype variation in response to warming. These plants showed a pre-adaptation of genes in anticipation of stress and the exclusive induction of genes involved in epigenetic mechanisms. Shallow plants also showed a stronger activation of heat-responsive genes and the exclusive activation of genes involved in molecular mechanisms that are behind their higher photosynthetic stability and respiratory acclimation.

## Author contributions

LM, JR, and GP planned and designed the study. LM and ED performed the heat-stress experiment, samples processing and lab work. LE conducted bioinformatics and data analysis. LM led the writing of the paper with contributions from the rest of authors. All Authors reviewed the manuscript.

### Conflict of interest statement

The authors declare that the research was conducted in the absence of any commercial or financial relationships that could be construed as a potential conflict of interest.
