# Supplementary material for: Molecular Mechanisms behind the Physiological Resistance to Intense Transient Warming in an Iconic Marine Plant
Source: Front Plant Sci. 2017 Jun 29;8:1142. doi: 10.3389/fpls.2017.01142 (PMC5489684; doi:10.3389/fpls.2017.01142)
Supplement: Supplementary file 4 [file DataSheet4.DOC]

**Supporting information**

**Molecular mechanisms behind the physiological resistance to intense transient warming in an iconic marine plant**

Marín-Guirao L.^1*^, Entrambasaguas L.^1^, Dattolo E.^1^, Ruiz J.M. ^2^, Procaccini G.^1^.

^1^Integrative Marine Ecology, Stazione Zoologica Anton Dohrn, Villa Comunale, 80121 Napoli, Italy.

^2^ Seagrass Ecology Group, Oceanographic Center of Murcia, Spanish Institute of Oceanography C/ Varadero, 30740 San Pedro del Pinatar, Murcia, Spain

*Corresponding author: Lazaro Marín-Guirao, tel. +39 081 583 3363, fax +39 081 764 1355, e-mail: [maringuirao@gmail.com](mailto:maringuirao@gmail.com)

**Appendix S1. Description of the results derived from the sequencing, *de novo* assembly and annotation of the transcriptome**

*Transcriptome sequencing, de novo assembly and annotation*

This is the first transcriptome of *Posidonia. oceanica* under short-term heat stress and a subsequent recovery period providing a valuable resource and a first step towards a comprehensive understanding of genome-wide gene expression of this relevant seagrass species, and by extension, of other aquatic plants

In this study, in the absence of a reference genome for *P. oceanica,* we *de novo* assembled a transcriptome to be used as a reference for read mapping and gene expression profiling after a 5 days exposure to an increased temperature of 32ºC and a subsequent 5-d recovery period under control conditions (24ºC). mRNA was isolated from leave tissues of three shallow and three deep *P. oceanica* genotypes in the control and the heat treatments after the heat exposure and recovery periods giving place to a total of 24 samples (2 depths x 2 treatments x 2 periods x 3 genotypes).

An overall profile of the transcriptome of *P. oceanica* leaves samples was obtained through Illumina sequencing of 24 cDNA libraries. 48 FASTQ files with a total of 776,971,548 paired-end reads of 101 bp were generated (Table SR1). FastQC overview of raw sequences showed that mean per-base quality remains above 30 for the first 99 bp (data not shown).

**Table AS1.** *Number of reads per library and in total (including the retained percentage) in each preprocessing step: raw reads, after rRNA removal and after quality trimming and filtering. The 24 libraries corresponded to the 24 leaf samples analyzed in the experiment (2 periods x 2 depth x 2 treatments x 3 replicates). D = deep and S = shallow.*

After quality trimming, filtration and rRNA removal, a total of 697,043,338 high quality paired-end reads (89.7% of initial raw reads) from all libraries were merged and provided as input to Trinity assembler. Transcriptome assembly resulted in 222,548 putative transcripts (no less than 200 bp) with an overall size of 390 Mb, an average length of 918 bp and GC percent of 44.5% (Table SR2). Filtering based on read abundance and component isoform percentage removed 86,602 sequences, leaving 135,946 isoforms (and 72,169 Trinity 'genes') with an overall size of 224 Mb, an average length of 984 bp and GC percent of 41.2% (Table SR2). On average, 82.7% of the reads mapped back to the assembly indicating that the assembly represented the majority of sequenced reads and thus, that the transcriptome was a reliable reference.

**Table AS2.** *Transcriptome assembly statistics of extensive and filtered de novo transcriptomes of P. oceanica.*

|  | **Extensive** | **Filtered** |
| --- | --- | --- |
| **Number of transcripts** | 222,548 | 135,946 |
| **Number of Trinity 'genes'** | 104,350 | 72,169 |
| **Average GC content (%)** | 44.53 | 41.19 |
| **N50** | 1,886 | 2,319 |
| **Mean (bp)** | 918.21 | 983.97 |
| **Median (bp)** | 427 | 384 |
| **Minimum contig length (bp)** | 224 | 224 |
| **Maximum contig length (bp)** | 17,138 | 17,138 |

Transcript length distribution analysis revealed more than 60.8% of the transcripts length less than 1,200 bases (42.8% of the transcript length less than 600 bases). This is a typical observation of most of the de novo transcriptome assemblies using Illumina short read deep sequencing approach (Fig. SR1).


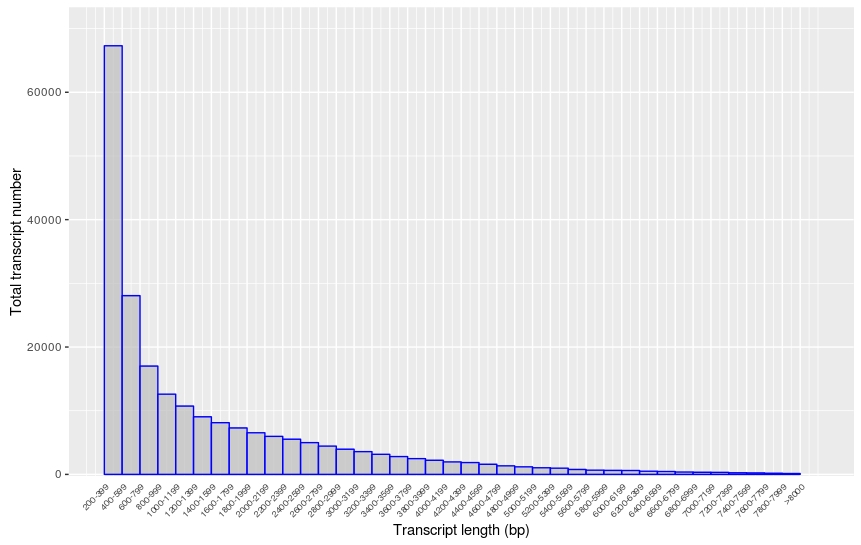


**Figure AS1**. *Size distribution of assembled transcripts identified from P. oceanica transcriptome.*

Similarity with known proteins (<E-values 1×10^-6^) was detected for 131,217 contigs which represent 58.96% of the *de novo* transcriptome assembly. Although most of the successfully annotated transcripts matched to the plant species Vitisvinifera (154,294, 69.33% of the total transcripts), *Elaeisguinensis* (108,977, 48.97%) and *Phoenix dactylifera* (102,704, 46.15%), most of the top blast hits (lowest e-value matching) matched to the seagrass *Zostera marina* (23.05% of the total top hits matches) (Fig. SR2) followed a long way behind by other plant species as *Elaneisguineensis* (12.45%) and *Phoenix dactylifera* (11.09%).


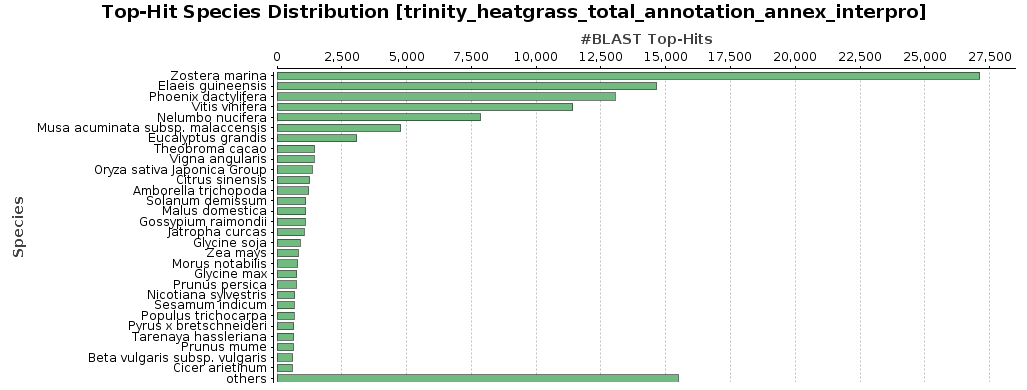


**Figure AS2**. *Number of top blast hits (lowest e-value matching) of successfully annotated transcripts of the novo assembled transcriptome of P. oceanica*.

In addition to the sequence similarity searches, we performed further functional annotation of the *P. oceanica* assembled transcripts to collect clues as much as possible to infer gene function. Based on sequence homology, 67,873 sequences (30.5% of the total transcripts) were successfully assigned to at least one GO term annotation; 22,709 sequences (10.2%) which retrieved GO terms associated with the hits obtained after a BLAST search but that did not surpass the annotation threshold and 40,300 sequences (18.11%) that had hits in the databases but lack functional information. A total of 348 GO terms were retrieved at level 3 where 50%, 22.99% and 27.01% of the terms were assigned to the biological process, molecular function and cellular component categories, respectively.

**Table S1.** Scores of the 12 microsatellite markers used for genotyping the experimental shallow (S) and deep (D) *P. oceanica* plants used in this study.

*Alberto et al., 2003; ** Procaccini and Waycott 1998.

Alberto F, Correia L, Arnaud-Haond S, Billot C, Duarte CM, Serrão E (2003) New microsatellite markers for the endemic Mediterranean seagrass *Posidonia oceanica*. *Molecular Ecology Notes,* **3**, 253-255.

Procaccini G, W aycott M (1998). Microsatellite loci identified in the seagrass *Posidonia oceanica* (L.) Delile. Journal of Heredity, **89**,562-568.

**Table S2.** List of primers (sequence 5’ to 3’) used for validation genes expression profiles.

**Figure S1.** Hierarchical clustering of differentially expressed genes (FDR<0.05) in heated shallow (S) and deep (D) P. oceanica plants at the end of the heat exposure and recovery periods. Heated and control samples are represented in red and blue letters respectively.

**
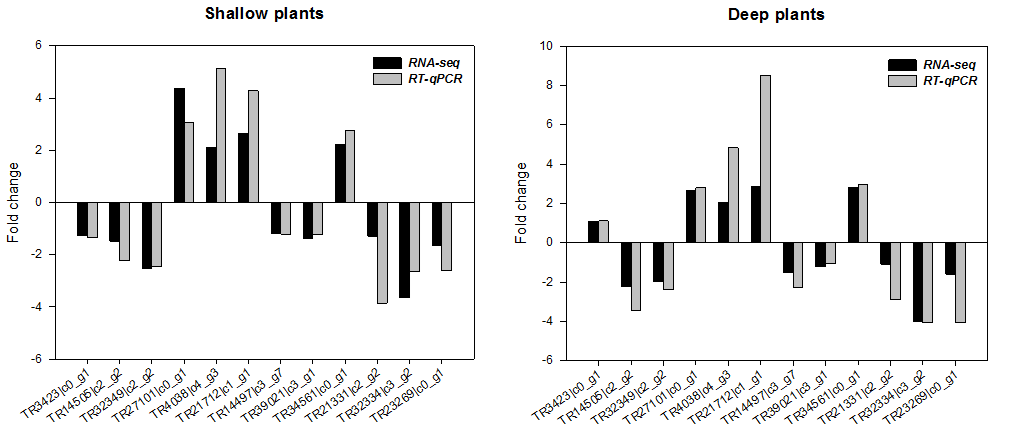
**

**Figure S2.** *Fold changes measured by RNA-Seq and RT-qPCR in shallow and deep P. oceanica genotypes*.
